# Effects of Unidirection/Bidirection Torsional Thermomechanical Processes on Grain Boundary Characteristics and Plasticity of Pure Nickel

**DOI:** 10.3390/ma15010236

**Published:** 2021-12-29

**Authors:** Yao Lin, Shan Liu, Tao Wu, Guangchun Wang

**Affiliations:** Key Laboratory for Liquid-Solid Structural Evolution and Processing of Materials (Ministry of Education), Shandong University, Jinan 250061, China; linyaoqcs@163.com (Y.L.); liushan890925@163.com (S.L.)

**Keywords:** pure nickel, unidirectional/bidirectional torsion, fraction of special boundary, plasticity

## Abstract

The “torsion and annealing” grain boundary modification of pure nickel wires with different diameters was carried out in this paper. The effects of torsional cycles as well as unidirectional/bidirectional torsion methods on grain boundary characteristic distribution and plasticity were investigated. The fraction of special boundaries, grain boundary characteristic distributions and grain orientations of samples with different torsion parameters were detected by electron backscatter diffraction. Hardness measurement was conducted to characterize the plasticity. Then, the relationship between micro grain boundary characteristics and macro plasticity was explored. It was found that the special boundaries, especially Σ3 boundaries, are increased after torsion and annealing and effectively broke the random boundary network. The bidirectional torsion with small torsional circulation unit was the most conducive way to improve the fraction of special boundaries. The experiments also showed that there was a good linear correlation between the fraction of special boundaries and hardness. The plasticization mechanism was that plenty of grains with Σ3 boundaries, [001] orientations and small Taylor factor were generated in the thermomechanical processes. Meanwhile, the special boundaries broke the random boundary network. Therefore, the material was able to achieve greater plastic deformation. Moreover, the mechanism of torsion and annealing on the plasticity of pure nickel was illustrated, which provides theoretical guidance for the pre-plasticization of nickel workpieces.

## 1. Introduction

Pure nickel has excellent corrosion resistance and high-temperature performance. It is widely used as a good structural material for high-precision mechanical products and electronic devices in industry, such as the battery poles and guiding wires in luminous parts [1,2]. With the booming development of precision manufacture, product miniaturization has become a new focus. It puts forward the higher requirements for the comprehensive mechanical properties, especially the plasticity of fine billets [3]. However, the production of micro products still suffers from defects such as inadequate precision and surface cracks due to poor plasticity. Therefore, rational material plasticizing ways are urgently needed in the pre-treatment of micro dimension billets.

In polycrystalline materials, the grain boundary (GB) controls many properties of materials such as hardness [4,5,6], intergranular corrosion [7,8,9] and GB fracture [10]. The “GB design and control” concept was primitively proposed in 1984 by T. Watanabe [11] and then developed into “grain boundary engineering (GBE)”, that is, increasing the fraction of low coincidence site lattice (low-ΣCSL) and optimizing the grain boundary characteristic distribution (GBCD) through rational deformation and heat treatment. The final objective is improving the material properties which are affected by GBs. The Σ value reflects the coincident degree of atoms on the GB. A smaller Σ means more coincident positions, lower GB energy and higher mobility [12], which results in some special variations in material properties. Therefore, the low-ΣCSL boundaries are also called special boundaries (SPs). SPs must satisfy at least two conditions: firstly, it is a CSL boundary with the Σ ≤ 29; secondly, the maximum misorientation deviation should under Brandon criterion. Both the CSL boundary with a Σ > 29 and the non-CSL boundary are referred to as random boundary [13].

Appropriate thermomechanical process (TMP) on FCC metals with low to medium stacking fault energy can stimulate the formation of SPs [12,13,14,15]. It has been proved that SPs have significant impacts on optimizing material properties. However, the mechanism of GB evolution is still under investigation. Models such as “Σ3 regenerating model” [16] and “migration and interaction of incoherent Σ3 model” [17] only can explain some experimental phenomena. It is worth noting that the significance of the fraction of SPs (*F*_sp_) and random boundary network connectivity are widely recognized. The optimizing by GBE can be manifested clearly only when the *F*_sp_ is high [18]. C. A. Schuh et al. [19] illustrated that grain clusters were generally irregularly shaped in nickel-based alloy. The large cluster size represented the bad random boundary connectivity. Afterwards, S. Xia et al. [20] proved that the formation of large grain clusters was an important indicator of the increase in SPs. Only the *F*_sp_ value alone was not sufficient to predict or reflect the random boundary network connectivity directly, as explained by B. M. Guyot et al. [12]. Both *F*_sp_ and boundary network connectivity should be investigated and jointly characterize the result of GB regulation. According to the study of grain clusters in Inconel 600 alloy [19], the evaluation indicator of GBCD should be considered not only the *F*_sp_, but also the blocking degree of random boundary network and the grain cluster size. In addition, the number of twins was found to increase with the growing grain size during annealing [21].

At present, the material optimizing via GB modification mainly focuses on improving the corrosion resistance and preventing intergranular crack. Research on promoting plasticity is insufficient and limited by the deformation method. Ru et al. [22] employed cold rolling and annealing to control the GB of the cupronickel alloy, increasing the *F*_sp_ to more than 75%. Unidirectional rolling and cross-rolling followed by annealing were performed on Incoloy 800 H/HT to optimize GBCD. Meanwhile, the differences of Σ3*^n^* (twin variation) boundary ratios and boundary interaction methods between these two rolling processes were analyzed [13].

In this paper, the “torsion and annealing” grain boundary modification of pure nickel wires with different diameters and torsional modes was carried out. The influences of torsional cycles, unidirectional/bidirectional torsion methods on the GBCD and plasticity were explored. The *F*_sp_, GBCD, grain size and grain boundary network connectivity of samples were characterized. Vickers hardness was employed to represent the plasticity. The relationship between micro grain boundary characteristics and macro plasticity was also investigated.

## 2. Materials and Methods

**Figure 1:**
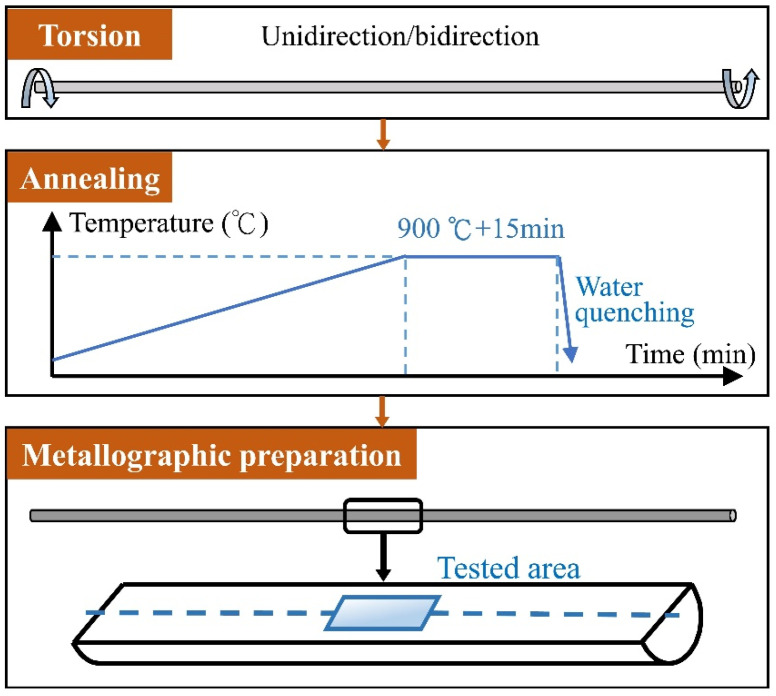
Schematic diagrams of the TMP and the metallographic preparation.

Commercial pure nickel Ni-200 (Ni > 99.6%) was chosen in this paper. The sample dimensions were φ1 mm × 160 mm and φ2 mm × 260 mm, respectively. φ indicated the diameter of the sample. As-received samples were all solution-annealed at 800 °C for 30 min to homogenize the microstructure and remove forming defects generated in the previous production. Subsequently, ZCXC-3 metal wire torsion tester was employed to conduct unidirectional and bidirectional torsion on solute samples. The torsional speed was 60 r/min. The detailed sample numbers and corresponding deformation parameters are listed in Table 1. Then, annealing treatment was carried out in NBD-T1500 vacuum tube furnace at 900 °C for 15 min on all torsional samples. The longitudinal section along central axis of the middle part of sample was selected for the microstructural analysis and mechanical test. Schematic diagrams of the TMP and the tested area are shown in Figure 1.

Rough grinding and mechanical polishing were conducted on all samples to obtain the observation surfaces. Subsequently, samples were electro-polished in the solution of 30% H_2_O + 70% H_2_SO_4_ with 12 V direct current for 12 s at room temperature. The electro-polish technique could remove residual stress of the observation surfaces. Then, samples were analyzed by a JSM-7800F scanning electron microscope equipped with an electron backscatter diffraction (EBSD) instrument set. Each scanning area was 600 μm × 400 μm and the step size was 1 μm. The crystallographic orientation data were analyzed in CHANNEL 5 and MATLAB software. In this research, the GB with a misorientation greater than 15° was recognized as a high-angle boundary. Brandon criterion was used to identify the CSL boundaries [23]. Grain size was measured by the linear intercept method. Vickers hardness tester HVS-1000A was employed to do hardness tests with a load of 0.5 kg and a dwell time of 10 s. For each sample, at least 5 hardness indentations were obtained, and the average value was taken as the final result. Tensile tests were conducted by a CMT5105 electronic universal material testing machine at room temperature. The gauge length was 100 mm, and the tensile speed was 2 mm/min. Each group of tensile tests was repeated 3 times to reduce the error.

## 3. Results and Discussion

### 3.1. Micro GB Characteristics

**Figure 2:**
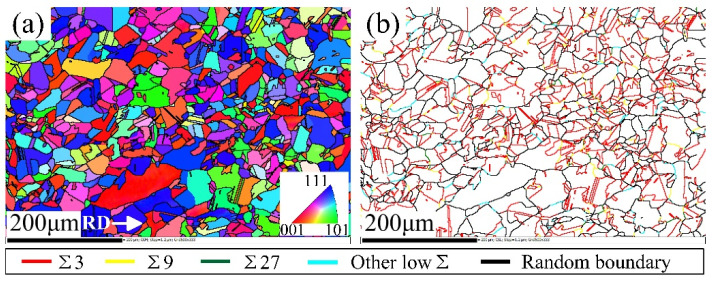
The (**a**) orientation imaging microscopy and (**b**) GBCD of the solution annealed sample with a diameter of 2 mm.

**Figure 3:**
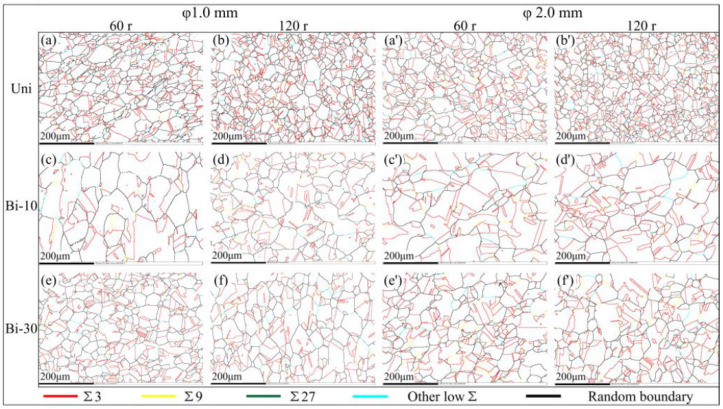
GBCD of samples: (**a**) φ1-Uni-1, (**b**) φ1-Uni-2, (**c**) φ1-Bi-10-1, (**d**) φ1-Bi-10-2, (**e**) φ1-Bi-30-1, (**f**) φ1-Bi-30-2, (**a’**) φ2-Uni-1, (**b’**) φ2-Uni-2, (**c’**) φ2-Bi-10-1, (**d’**) φ2-Bi-10-2, (**e’**) φ2-Bi-30-1, (**f’**) φ2-Bi-30-2.

Figure 2 shows the orientation imaging microscopy and GBCD of the solution annealed sample with a diameter of 2 mm. The microstructure of the φ1 mm sample after solution annealing is very similar to above φ2 mm sample. Figure 3 shows the GBCD of samples with various parameters after thermomechanical treatment. The types of grain boundaries are represented by different colors, as shown in the legend. A large number of SPs, many grain clusters and random boundary networks broken by SPs could be observed. Among all SPs, Σ3 boundaries accounted for the most and were evenly distributed. Few other CSL boundaries were scattered in the material.

**Figure 4:**
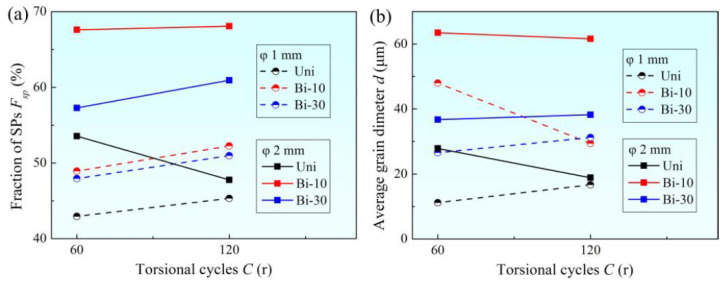
Variations of (**a**) *F*_sp_ and (**b**) *d* with torsional cycles.

The variations of *F*_sp_ and average grain size *d* with torsional cycles in samples are shown in Figure 4a,b. In this paper, low angle boundaries were not considered, and the *F*_sp_ referred to the length ratio of SPs to the total high-angle boundaries. The *F*_sp_ values of φ1 mm and φ2 mm samples after solution annealing were 49.20% and 41.39%, respectively, and the grain sizes were 21.84 μm and 25.01 μm, respectively. As shown in Figure 4a, in the φ2 mm sample, it was easier to obtain a large *F*_sp_ value of 68.08%, which was much higher than the highest *F*_sp_ (52.25%) of the φ1 mm sample. Mostly, the *F*_sp_ value of a sample with the total torsional cycles of 120 r was higher than that of the total torsional cycles of 60 r under the same torsional mode. For the same total torsional cycles, the *F*_sp_ value under the condition of unidirectional torsion was obviously low. However, the *F*_sp_ obtained by bidirectional torsion with small circulation unit (10 r) and multiple circulations was the highest. The variations of average grain diameter and *F*_sp_ value with torsional mode were not very consistent. In general, the average grain size of φ2 mm sample was larger than that of φ1 mm sample. The grains of unidirectional torsion were also generally smaller than those of bidirectional torsion under the same other conditions, especially when using the small torsional circulation unit (10 r).

**Figure 5:**
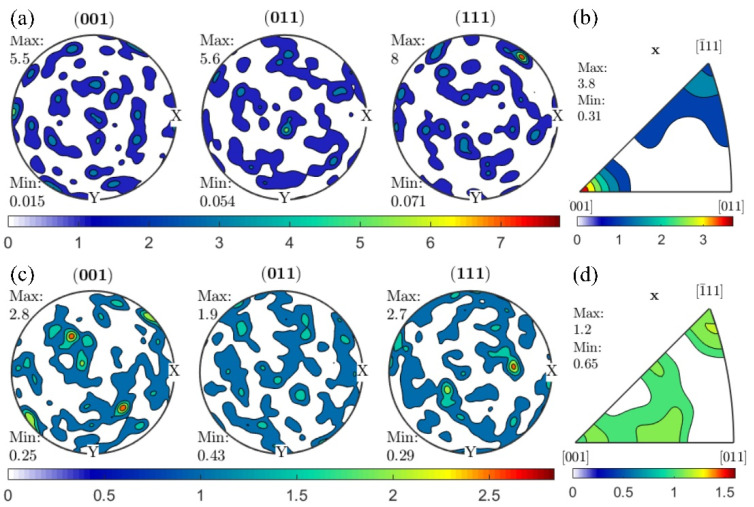
Pole figures of samples: (**a**) φ2-Bi-10-1 and (**c**) φ1-Uni-2. Inverse pole figures of samples: (**b**) φ2-Bi-10-1 and (**d**) φ1-Uni-2. The x direction indicates the axial direction of the sample.

The pole figures and inverse pole figures of samples φ2-Bi-10-1 and φ1-Uni-2 were displayed in Figure 5. The φ2-Bi-10-1 has much higher *F*_sp_ value and bigger grains than φ1-Uni-2. It could be seen from the pole figures that the pole points were uniformly distributed, and there was no obvious deformation texture in both samples, indicating that they had basically recrystallized. However, the inverse pole figures revealed that there were still some preferred orientations in the materials. In sample φ2-Bi-10-1, the grain orientations tended to align along the [001] direction (recrystallization texture). Newly generated Σ3 boundaries did not necessarily lie on their ideal planes of (111) or (112), which was consistent with the research by H.M. Miller [24]. The preferred orientation [001] in sample φ2-Bi-10-1 was likely to be related to the large amount of Σ3 twin boundaries which were formed in recrystallization. An orientation of [001] might be more conducive to the formation of annealing twins and the generation of Σ3 boundaries [25]. However, no preferred crystal orientation existed along axial direction in sample φ1-Uni-2.

### 3.2. Vickers Hardness

**Figure 6:**
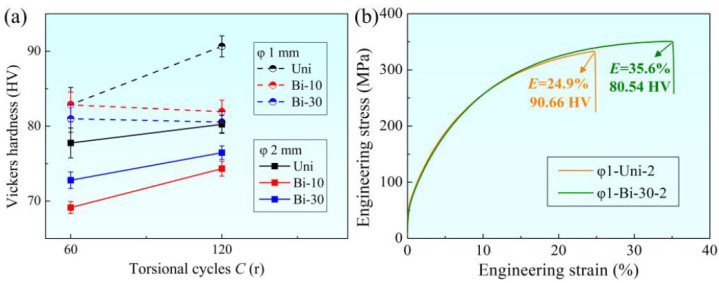
(**a**) Variations of Vickers hardness with torsional cycles. (**b**) The strain–stress curves of samples φ1-Uni-2 and φ1-Bi-30-2.

The variations of Vickers hardness of samples with different torsional modes and the same heat treatment are shown in Figure 6a. In order to better characterize the plasticity, uniaxial tensile tests at room temperature were carried out on φ1 mm samples with the highest and lowest hardness. The corresponding stress–strain curves are shown in Figure 6b. It can be seen from Figure 6 that the characterization of plasticity by elongation and hardness are consistent. It was found that the hardness of the φ2 mm sample was generally lower than that of the φ1 mm sample. For the φ2 mm sample, the hardness was significantly higher when the total torsional cycles were large or in the condition of unidirectional torsion. However, for the bidirectional torsion of the φ1 mm sample, the hardness decreased with the increase in total torsional cycles. Therefore, the deformation mode of bidirectional torsion was more conducive to improving the plasticity.

### 3.3. Relationship between Microstructure Characteristics and Plasticity

**Figure 7:**
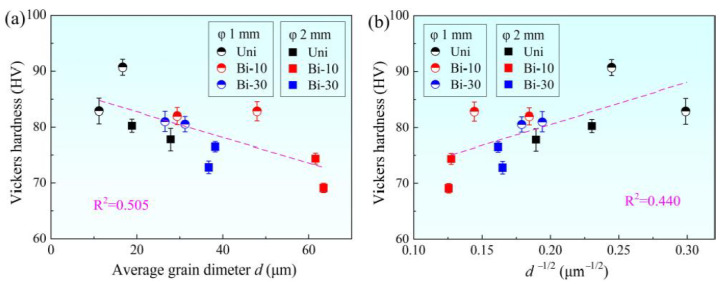
Relationship between Vickers hardness with (**a**) *d* and (**b**) *d*^−1/2^.

In order to explore the effect of grain size on hardness of samples obtained by different torsion methods, the relationship between grain size *d* and Vickers hardness is shown in Figure 7. With the increase in grain size *d*, Vickers hardness generally declined. However, there was no clear linear correlation between them. The goodness of fit R^2^ was only 0.505. According to the Hall–Petch relationship, the hardness is inversely proportional to the square root of grain diameter. Take Vickers hardness as a function of *d*^−1/2^, as shown in Figure 7b. It was found that the linear relationship between these two variables was still unclear, and the goodness of fit R^2^ was as low as 0.440. This proved that the relationship between grain size and hardness could not be considered simply from the perspective of the Hall–Petch formula, which was consistent with the former research [26]. On one hand, in the theoretical model, the smaller the grain size, the higher the grain boundaries were in equal-volume polycrystalline materials. Meanwhile, the grain boundary strength was higher than the intracrystalline strength. More boundaries led to higher strength and hardness. On the other hand, the smaller the grain size, the higher the grain numbers, which brought about better coordination in plastic deformation. Thus, the material could withstand larger plastic deformation. In conclusion, the grain size was unable to well reflect the Vickers hardness or plasticity of samples after grain boundary regulation.

**Figure 8:**
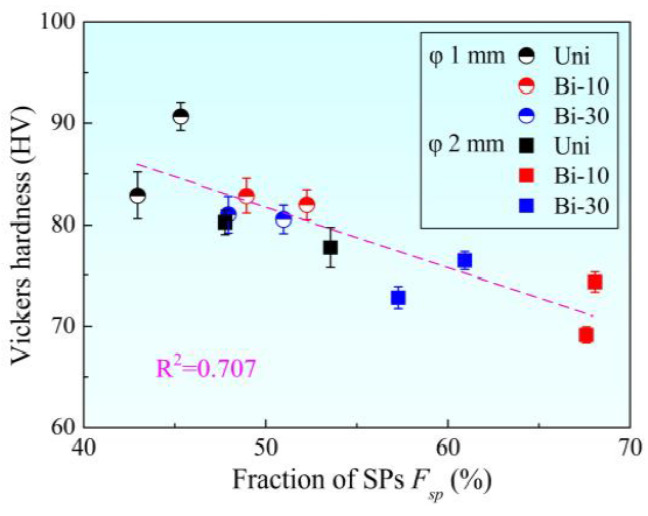
Relationship between Vickers hardness and *F*_sp_ value.

The relationship between micro grain boundary characteristic and Vickers hardness was further explored. Vickers hardness was expressed as a function of the *F*_sp_ value, as shown in Figure 8. It could be seen from the figure that there was a closer linear correlation between *F*_sp_ and Vickers hardness than that of the grain size. Vickers hardness decreased with the increase in *F*_sp_. This was because SPs, especially the numerous Σ3 boundaries, could inhibit the crack initiation and propagation [27,28,29,30,31]. Furthermore, Σ3 boundaries showed weaker hindrance to dislocation slip compared with other grain boundaries, leading to more uniform strain distribution in materials. On the contrary, crack was more likely to occur, and the propagation rate was faster at random boundaries [10]. Therefore, the higher the *F*_sp_ value, the lower the hardness and the better the plasticity.

**Figure 9:**
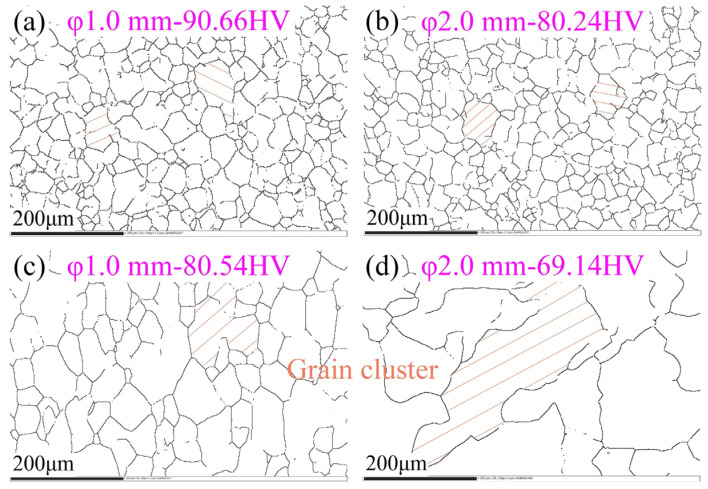
Random boundary distributions corresponding to the maximum and minimum hardness of samples with different diameters: (**a**) φ1-Uni-2, **(b**) φ2-Uni-2, (**c**) φ1-Bi-30-2, (**d**) φ2-Bi-10-1.

The damage degree of random boundary network is also worth noting. The essential reason for the improvement of material properties by grain boundary modification is that the numerous SPs effectively broke the random boundary network, which was easy to fracture or be corroded. Therefore, the material could have strong resistance to intergranular cracking and corrosion. The random boundary distributions corresponding to the maximum and minimum hardness of TMP samples with different diameters are shown in Figure 9. It can be seen from the figure that the sizes of grains and grain clusters of high hardness samples were small, and the random boundary networks were complete. However, for samples with low hardness, it was obvious that the random boundary networks were seriously damaged and no longer continuous. In addition, the grains and grain clusters were very large, especially for sample φ2-Bi-10-1, of which the grain cluster size could be up to hundreds of microns, as shown in Figure 9d. According to the previous analysis, in this case, the microcracks generated in the process of plastic deformation were difficult to propagate in materials, while the dislocation slipping was more unimpeded. Therefore, the more serious the damage of random boundary network, the better the plasticity.

**Figure 10:**
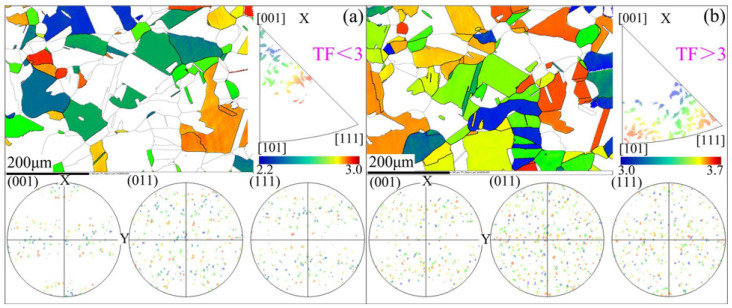
Taylor factor distribution map; corresponding pole figures and inverse pole figures of sample φ2-Bi-10-1: (**a**) TF < 3 and (**b**) TF > 3.

Taylor factor (TF) is a reliable index to characterize plasticity. TF represents the ability of a crystal to resist plastic deformation. The smaller the TF, the less slipping is required for deformation. Thus, the grain with a lower TF is often considered to be softer than the grain with a higher TF [32]. Now, analyze the TF of sample φ2-Bi-10-1 together with its pole figure and inverse pole figure, as illustrated in Figure 10. Figure 10a shows the distribution of TFs which are less than 3. It was found that the grain orientations with smaller TFs tended to align toward the [001] direction. On the contrary, the grain orientations with larger TFs were more likely to lie along the [011] and [111] direction (Figure 10b). Therefore, it could be inferred that the [001] orientation of grains was more conducive to generating plastic deformation. Combined with the analysis in Section 3.1, it could be known that in a [001] dominated situation, and Σ3 twin boundaries were easy to form with small TFs, leading to the better plasticity of materials.

## 4. Conclusions

Nickel wires with different diameters were subjected to “torsion and annealing” grain boundary modification to study the effects of torsional parameters on the GBCD and plasticity. The micro characteristics related to GBs were investigated, and Vickers hardness was employed to characterize the plasticity. Then, the relationship between micro characteristics and plasticity was illustrated, as well as the plasticizing mechanism. This work provides theoretical guidance for the pre-plasticization of nickel materials. Moreover, it is of great significance to improve the forming quality of complex nickel parts. The main findings are as follows:

1. The SPs, especially Σ3 boundaries, increased and effectively broke the random boundary networks after “torsion and annealing” thermomechanical treatment. Samples with a diameter of 2 mm generally had higher fraction of SPs, larger average grain size and lower hardness than that of the 1 mm diameter sample. The *F*_sp_ and average grain size were higher by bidirectional torsion than those by unidirectional method, whereas the hardness was much lower. The bidirectional torsion with small torsional circulation unit (10 r) was the most appropriate way to improve the fraction of SPs.
2. Vickers hardness of nickel samples decreased with the increase in the *F*_sp_. That is, there was a good linear correlation between *F*_sp_ and hardness. No clear regularity existed between grain size and hardness.
3. The plasticization mechanism of thermomechanical treated nickel wires was as follows. On one hand, numerous Σ3 boundaries broke the random boundary network, which was easy to fracture, and hindered the crack initiation and propagation. On the other hand, the Σ3 annealing twin boundaries formed in thermomechanical treatment were stimulated by a [001] dominated orientation distribution which had low TF. Thus, the hardness was reduced and the plasticity of materials was improved.

## Figures and Tables

**Table 1:** Detailed sample numbers and corresponding deformation parameters.

Diameter [mm]	Positive Torsion Cycles [r]	Reverse Torsion Cycles [r]	Circulation Numbers	Total Cycles [r]	No.
1	60	-	1	60	φ1-Uni-1
	10	10	3		φ1-Bi-10-1
	30	30	1		φ1-Bi-30-1
	120	-	1	120	φ1-Uni-2
	10	10	6		φ1-Bi-10-2
	30	30	2		φ1-Bi-30-2
2	60	-	1	60	φ2-Uni-1
	10	10	3		φ2-Bi-10-1
	30	30	1		φ2-Bi-30-1
	120	-	1	120	φ2-Uni-2
	10	10	6		φ2-Bi-10-2
	30	30	2		φ2-Bi-30-2

Note: “Uni” represents unidirectional torsion and “Bi” represents bidirectional torsion.

## Data Availability

The raw/processed data required to reproduce these findings cannot be shared at this time as the data also form part of an ongoing study.
